# The Mutation Profile of Calreticulin in Patients with Myeloproliferative Neoplasms and Acute Leukemia

**DOI:** 10.4274/tjh.2015.0220

**Published:** 2016-08-19

**Authors:** Jingyi Wang, Jianguo Hao, Na He, Chunyan Ji, Daoxin Ma

**Affiliations:** 1 Qilu Hospital of Shandong University, Department of Hematology, Shandong, China; 2 Affiliated Hospital of Shandong University of Traditional Chinese Medicine, Department of Hematology, Shandong, China; 3 General Hospital of Shandong Stell Group Company, Department of Surgery, Shandong, China

**Keywords:** Calreticulin mutation, Myeloproliferative neoplasms, Leukemia

## Abstract

**Objective:**

Calreticulin (CALR) plays important roles in cell proliferation, apoptosis, and immune responses. CALR mutations were described recently in Janus kinase 2 gene (JAK2)-negative or MPL-negative primary myelofibrosis (PMF) and essential thrombocythemia (ET) patients. CALR trails JAK2 as the second most mutated gene in myeloproliferative neoplasms (MPNs). However, little is known about CALR mutation in Chinese patients with leukemia. In the present study, a cohort of 305 Chinese patients with hematopoietic neoplasms was screened for CALR mutations, with the aim of uncovering the frequency of CALR mutations in leukemia and MPNs.

**Materials and Methods:**

Polymerase chain reaction and direct sequencing were performed to analyze mutations of CALR in 305 patients with hematopoietic malignancies, including 135 acute myeloid leukemia patients, 57 acute lymphoblastic leukemia patients, and 113 MPN patients.

**Results:**

CALR mutations were found in 10.6% (12 of 113) of samples from patients with MPNs. CALR mutations were determined in 11.3% (6 of 53), 21.7% (5 of 23), and 9.1% (1/11) of patients with ET, PMF, and unclassifiable MPN, respectively.

**Conclusion:**

We showed that MPN patients carrying CALR mutations presented with higher platelet counts and lower hemoglobin levels compared to those with mutated JAK2. However, all of the leukemia patients had negative results for CALR mutations.

## Abstract (tr)

**Amaç:**

Kalretikülin (CALR) hücre çoğalması, apoptoz ve immün yanıtlarda önemli rol oynar. CALR mutasyonları yakın zamanda Janus kinaz 2 (JAK2) veya MPL geni negatif primer miyelofibroz (PMF) ve esansiyel trombositemi (ET) hastalarında tanımlanmıştır. CALR JAK2’yi takiben miyeloproliferatif neoplazilerde (MPN) ikinci sıklıkta görülen mutant gendir. Ancak, Çinli lösemi hastalarında CALR mutasyonları hakkında bilgi sınırlıdır. Bu çalışmada, hematopoetik neoplazisi olan 305 Çinli hasta CALR mutasyonları, bu mutasyonların lösemi ve MPN hastalarındaki sıklığının ortaya çıkarılması için taranmıştır.

**Gereç ve Yöntemler:**

Polimeraz zincir reaksiyonu ve direkt dizileme yöntemi 135 akut miyeloid lösemi, 57 akut lenfoblastik lösemi ve 113 MPN olmak üzere toplam 305 hematopoetik malinitesi olan hastada CALR mutasyonlarını analiz etmede kullanılmıştır.

**Bulgular:**

CALR mutasyonu MPN hastalarının %10,6’sında (12/113) tespit edilmiştir. Ayrıca bu mutasyonlar ET, PMF ve sınıflandırılamayan MPN hastalarında sırasıyla %11,3 (6/53), %21,7 (5/23) ve %9,1 (1/11) olarak bulunmuştur.

**Sonuç:**

CALR mutasyonu taşıyan MPN hastaları JAK2 pozitif olanlara göre tanı anında daha yüksek trombosit sayısı ve daha düşük hemoglobin düzeylerine sahip olduklarını gösterdik. Ancak, lösemi hastalarının tamamında CALR mutasyonları negatif tespit edildi.

## INTRODUCTION

Somatic frameshift mutations in exon 9 of calreticulin (CALR) have been identified in a large proportion of JAK2- or MPL-negative myeloproliferative neoplasm (MPN) patients, including those with primary myelofibrosis (PMF) and essential thrombocythemia (ET) [1,2]. The CALR gene, located on chromosome 19p13.3, encodes a 48-kDa protein that consists of three domains: the amino terminal N-domain (residues 1-180), central proline-rich P-domain (residues 181-290), and carboxyl terminal C-domain (residues 291-400). The CALR protein is localized primarily in the endoplasmic reticulum through its C-terminal KDEL motif [3], but it is also found in the cell membrane, cytoplasm, and extracellular matrix [4,5]. Functionally, CALR is believed to participate in Ca2+ homeostasis as a calcium-binding protein, handling misfolded proteins, cell adhesion, immune response to cancer, and phagocytosis [4,6,7,8,9,10,11,12,13]. CALR-knockout mice are born dead and display impaired cardiac development, whereas postnatal overexpression also leads to cardiac defects [14,15]. Therefore, CALR regulates key cellular functions like proliferation and apoptosis. CALR also plays an important role in immune responses [16].

Mutations of CALR were found essential for the diagnosis and prognosis of MPNs in recent years. All CALR mutations seen so far in MPNs mainly involve exon 9 and are somatic insertions or deletions. Two mutation variants (type 1 and type 2) were the most frequent: type 1 (c.1179_1230del) resulted from a 52-bp deletion, more frequent in PMF, and type 2 (c.1234_1235insTTGTC) resulted from a 5-bp TTGTC insertion [1]. Andrikovics et al. demonstrated that CALR mutations are found in about one-fourth of patients with ET or PMF and are associated with distinct clinical characteristics, and another study also found that CALR mutations are associated with younger age, more severe anemia, higher white blood cell (WBC) and platelet counts, lower Dynamic International Prognostic Scoring System Plus scores, and better survival compared to subjects with JAK2 mutations [17,18].

Similar to MPNs, acute leukemia, including acute myeloid leukemia (AML) and acute lymphoblastic leukemia (ALL), is a group of disorders characterized by abnormal clonal proliferation and immune imbalance. To investigate whether CALR mutations were present in myeloid neoplasms, Andrikovics et al. detected JAK2, CALR, and MPL genes in 289 cases of ET and 99 cases of PMF, and they reported that in ET, 154 (53%) JAK2V617F mutation-positive, 96 (33%) CALR mutation-positive, 9 (3%) MPL mutation-positive, and 30 triple-negative (11%) cases were identified, while in PMF 56 (57%) JAK2V617F mutation-positive, 25 (25%) CALR mutation-positive, 7 (7%) MPL mutation-positive, and 11 (11%) triple-negative cases were identified [18]. Qiao et al. screened CALR mutations in 104 AML patients, 55 chronic myeloid leukemia (CML) patients, 7 chronic myelomonocytic leukemia patients, and 8 myelodysplastic syndrome (MDS) patients. Although most of these patients had negative results, one AML patient was found to harbor a CALR mutation (c.1179_1230del) without JAK2V617F or MPL W515L/K mutations [19].

Unlike AML, ALL is a heterogeneous malignancy caused by the clonal proliferation of lymphocytes. However, no data about the mutation frequency of CALR in ALL patients have been reported to date. Therefore, in the present study, a cohort of 305 Chinese patients with hematopoietic neoplasms was screened for CALR mutations, with the aim of uncovering the frequency of CALR mutations in leukemia and MPNs. The results demonstrate that CALR mutation status is an important diagnostic factor in MPN patients without JAK2 mutation while it is negative in leukemia patients.

## MATERIALS AND METHODS

### Subjects and Ethics Statement

Bone marrow or peripheral blood samples from 113 MPN patients were collected at Qilu Hospital of Shandong University between August 2012 and November 2014, including cases of ET (n=53), polycythemia vera (PV; n=20), PMF (n=23), MDS/MPN (n=6), and unclassifiable MPN (MPN-U; n=11). We also obtained bone marrow samples from 192 patients with other hematopoietic neoplasms including AML (n=135) and ALL (n=57). These patients were all newly diagnosed before treatment. The characteristics of the patients at the time of sampling are presented in Tables 1 and 2. The patients with AML were treated with standard induction chemotherapy (anthracycline and cytarabine). The patients with ALL were treated with standard induction chemotherapy (vincristine, daunorubicin, L asparaginase, and prednisone). Bone marrow mononuclear cells (BMMCs) or peripheral blood mononuclear cells (PBMCs) were obtained from patients using density-gradient centrifugation with the Ficoll-Hypaque technique (Ficoll, Pharmacia LKB Biotechnology Inc., Piscataway, NY, USA). The samples were then stored at -80 °C. The present study was approved by the Ethics Committee of Qilu Hospital, Shandong University (Jinan, China). Written informed consent was obtained from all participants for treatment and the cryopreservation of bone marrow and peripheral blood according to the Declaration of Helsinki.

### Genomic DNA Isolation, Polymerase Chain Reaction Amplification, and Sequencing

Genomic DNA samples from BMMCs or PBMCs of patients were extracted using the TIANGEN DNA Extraction Kit (TIANamp Genomic DNA Kit, Beijing, China). Oligonucleotide primers targeting exon 9 of CALR were used to amplify a 377-bp product: forward 5’ - CTG GCA CCA TCT TTG ACA ACT T - 3’, reverse 5’ - GGC CTC TCT ACA GCT CGT C - 3’. Polymerase chain reaction (PCR) was performed in a volume of 25 µL containing 150 ng of DNA, 12.5 µL of PCR master mix, 400 nM each of forward and reverse primers, and ddH2O. Cycling parameters consisted of an initial denaturation at 94 °C for 2 min; 40 cycles of denaturation at 94 °C for 15 s, annealing at 56 °C for 30 s, and extension at 72 °C for 45 s; and a final extension at 72 °C for 1 min. PCR products were purified (QIAquick PCR Purification Kit, QIAGEN, Valencia, CA, USA) and subjected to bidirectional sequencing. Mutations were identified using Mutation Surveyor Software (Soft Genetics, LLC, State College, PA, USA).

JAK2V617F mutation burden was assessed using a quantitative PCR-based allelic discrimination assay. Real-time quantitative PCR was conducted using an ABI Prism 7500 Real-Time PCR System (Applied Biosystems, Foster City, CA, USA) in accordance with the manufacturer’s instructions. The primers and probes were as follows: JAK2-PCR-Primer-F: AAG CTT TCT CAC AAG CAT TTG GTT T, JAK2-PCR-Primer-R: AGA AAG GCA TTA GAA AGC CTG TAG TT, MGB probe sequence: JAK2-Probe-WT: VIC- TCT CCA CAG ACA CAT AC; JAK2-Probe-V617F: FAM- TCC ACA GAA ACA TAC (all the primers and probes were synthesized by Invitrogen, USA). The real-time PCR contained, in a final volume of 10 µL, 1 µL of DNA, 5 µL of Universal PCR Master Mix, 0.4 µL of Primer-F and Primer-R, 0.2 µL of Probe-WT, 0.2 µL of Probe-V617F, and 2.8 µL of distilled water. PCR reaction was done at 50 °C for 2 min and 95 °C for 15 min, followed by 45 cycles of 95 °C for 30 s and 62 °C for 1 min. The fluorescence signal was collected at 62 °C while ROX Reference Dye was used as a background to normalize the fluorescent signal. The cycle threshold (Ct) value of VIC or FAM reflects the number of wild-type or mutant JAK2V617F gene DNAs, denoted as Ct VIC and Ct FAM. JAK2V617F was considered as positive when CtFAM was lower than 38.

### Statistical Analysis

The Kolmogorov-Smirnov test was performed to test whether variables were normally distributed. Then the independent-samples t-test was used to compare continuous variables. Chi-square or Fisher exact tests were used for dichotomous variables. The clinical characteristics of the leukemia and MPN patients, including sex, age, WBC count, and other factors, are presented in Tables 1 and 2. Statistical analysis was performed using SPSS 17.0 (SPSS Inc., Chicago, IL, USA).

## RESULTS

### The Profile of CALR Mutations in MPN Patients

**Figure 1:**
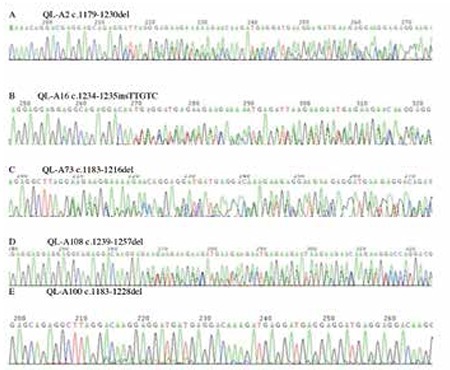
Sequencing results of CALR mutations in patients with myeloproliferative neoplasms.

Mutant CALR in MPNs is a result of frameshift mutations, caused by exon 9 deletions or insertions; the type 1 variant, a 52-bp deletion (c.1179_1230del), and type 2 variant, a 5-bp TTGTC insertion (c.1234_1235insTTGTC), constitute more than 80% of these mutations. In our study, a total of 10.6% of patients (12 of 113) with MPNs were demonstrated to harbor CALR mutations. The CALR mutation was found in 11.3% (6 of 53) of ET, 21.7% (5 of 23) of PMF, and 9.1% (1/11) of MPN-U patients, respectively (Table 3). Moreover, CALR mutations were found in 24.0% of JAK2V617F-negative ET patients (6 of 25) and 35.7% of JAK2V617F-negative PMF patients (5 of 14). No CALR mutation was found in patients with PV. For mutation types, a total of 5 distinct variants of CALR mutation, including 4 deletions and 1 insertion, were identified (Figure 1). c.1179_1230del, which resulted from a 52-bp deletion, and c.1234_1235insTTGTC, which resulted from a 5-bp insertion, were the most frequent CALR mutations. The two mutations accounted for 50% (6 of 12) and 25% (3 of 12) in all cases with mutant CALR, respectively. For ET patients, the two mutations were 50% (3 of 6) and 50% (3 of 6), respectively. For PMF patients, the two mutations were 60% (3 of 5) and 0% (0 of 5), respectively. Moreover, we also identified other kinds of deletions of CALR genetic variation: c.1239_1257del (1/12) and c.1183_1228del (1/12) were found in ET patients, and c.1183_1216del (1/12) was found in a MPN-U patient.

### The Profile of CALR Mutations in Leukemia Patients

**Figure 2:**
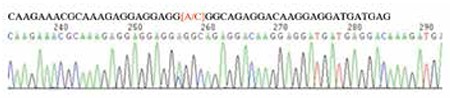
CALR rs143880510 single nucleotide polymorphism in one acute lymphoblastic leukemia patient.

To investigate whether CALR mutations were present in other hematopoietic neoplasms, we screened 135 patients with AML and 57 patients with ALL. However, no CALR exon 9 mutations were found in any of these patients. One single nucleotide polymorphism (SNP) of CALR, rs143880510 (Figure 2), was found in one ALL patient.

### Clinical Features of Patients with CALR Mutations

All of the 20 PV patients and 6 MDS/MPN patients had wild-type CALR. ET patients with mutant CALR had lower WBC counts (7.5±4.1×10^9^/L; p<0.001), lower hemoglobin levels (137±34.2 g/L; p=0.002), and higher platelet counts (982±24.2×10^9^/L; p<0.001) than those with mutant JAK2V617F (14±11.0×10^9^/L, 145±21.4 g/L, 515±31.6×10^9^/L). Similarly, PMF patients with mutant CALR showed lower hemoglobin levels (66.5±14.1 g/L; p=0.001) than mutant JAK2 patients (150±25.7 g/L; p=0.001).

ET patients with mutant CALR were significantly younger (44.0±15.1 years; p<0.001) than those with mutant JAK2 (56.2±12.9). No significant difference was identified between ET patients with mutant CALR and mutant JAK2 in terms of sex (Table 4). There was no significant difference in sex, age, WBC count, or platelet count between PMF patients with mutant CALR and mutant JAK2 (Table 4).

## DISCUSSION

Since the first description of myeloproliferative diseases by Dameshek in 1951 [20], there has been a consecutive progression in the understanding of these disease conditions characterized by abnormal bone marrow hyperplasia. Apart from the characterization of the Philadelphia chromosome in CML, the discovery of JAK2V617F mutation in 2005 [21,22] is the most thrilling development in the molecular diagnosis of Ph-negative MPNs. The subsequently reported somatic mutation in JAK2 exon 12 [23], though much less prevalent in the patients, is considered as another robust molecular marker for Ph-negative MPNs, and especially for PV patients.

The mutations in JAK2, MPL, and CALR are driver mutations, and they all activate the JAK2 pathway, but additional recurrent somatic mutations in several genes (TET2, ASXL1, DNMT3A, CBL, LNK, IDH1/2, IKF1, EZH2, TP53, SRSF2), encoding transcriptional and epigenetic regulators and signaling proteins, occur in MPNs. These additional mutations modulate disease progression and can also occur as primary mutations, but it is now convincingly demonstrated that MPNs can be initiated from a single JAK2V617F hematopoietic stem cell.

JAK mutations have also emerged in other hematologic diseases, and the majority of the pathogenic mutations in JAK2 (also in JAK1 and JAK3) localize in or near the pseudokinase domain.

To date, somatic frameshift mutations in exon 9 of CALR have been identified in a large proportion of JAK2- and MPL-negative PMF and ET patients. In a study of 617 PMF patients by Rumi et al., 399 (64.7%) carried JAK2V617F, 140 (22.7%) had a CALR exon 9 indel, 25 (4.0%) carried an MPL (W515) mutation, and 53 (8.6%) had nonmutated JAK2, CALR, and MPL (so-called triple-negative PMF) [24]. Kim et al. investigated mutation profiles of CALR, JAK2, and MPL in 199 Korean patients with MPNs. The overall frequency of CALR mutations was 12.6%; it was most frequent in MPN-U cases (37.5%), followed by ET (17.7%) and PMF (14.8%). CALR mutations were not found in PV or acute panmyelosis with myelofibrosis. CALR and JAK2 or MPL mutations were mutually exclusive [25]. Wu et al. also found two kinds of CALR mutations, c.1179_1230del and c.1234_1235insTTGTC, in Chinese patients with MPNs, and female patients showed a predisposition to CALR mutation [26]. Li et al. studied 1088 Chinese patients with MPNs including ET (n=234) and PMF (n=50) without JAK2V617F or MPL exon 10 mutations. CALR mutation was detected in 53% of subjects with ET and 56% of subjects with PMF, and 152 CALR mutations were identified clustering into 15 types, including deletions (n=8), insertions (n=3), and complex indels (n=4) [27].

In our study, mutations in CALR were present in 12 of 113 patients with Ph-negative MPNs (10.6%). Mean while, an overwhelming majority (75%) of the CALR mutation pattern still lies in c.1179_1230del and c.1234_1235insTTGTC. CALR mutations were present in 12 of 50 MPN patients without JAK2 mutations (24%). Among patients with ET, those with CALR mutations, as compared with those with JAK2V617F mutations, presented with significantly higher platelet counts and lower hemoglobin levels.

Whereas the frequency of CALR mutations in MPNs is quite consistent in recent studies, it is unclear whether CALR mutations occur in up to 8.3% of patients with MDS (10 of 120 MDS patients) as reported by Nangalia et al. [2], or are infrequent in MDS (none of 73) and AML (none of 254) patients as reported by Klampfl et al. [1]. This inconsistency could be due to the relatively small number of investigated patients. Therefore, in addition to MPNs, the mutation profile of CALR in other hematopoietic diseases such as leukemia and MDS has been given more attention than in the past. CALR mutations were identified in 2 of 527 MDS patients (0.38%). None of 328 patients with MDS were found to have CALR mutations. Two of 199 patients with AML following MDS had mutated CALR, and the frequency of CALR mutations is very low in MDS, supporting the use of CALR mutations as a diagnostic marker for ET and PMF patients [28]. Recently in a Chinese study, Cui et al. sequenced CALR mutations in 14 patients who met the WHO criteria for chronic neutrophilic leukemia (CNL) and found that 1 of 14 CNL patients had a CALR mutation (c.1154-1155insTTGTC) [29].

No CALR mutations were found in 62 patients with ALL [2]. However, little attention has been paid to AML and no data about the mutation frequency of CALR in Chinese ALL patients have been reported until now. Therefore, we screened 135 AML patients and 57 ALL patients. However, no CALR exon 9 mutations were found in any of these patients. Only one of the leukemia patients was found to have a CALR SNP, rs143880510.

To date, detection of CALR mutations in peripheral blood has been used as a diagnostic tool in the same way that tests for JAK2 mutations have simplified and improved the accuracy of diagnosis of patients with MPNs. However, in order to develop novel therapeutic drugs, further research is needed to explore the relationship between the pathogenesis of MPNs and the function of CALR.

## CONCLUSION

In summary, our data from this cohort of Chinese patients with MPNs confirmed that CALR mutations were novel molecular markers in JAK2V617F-negative MPNs. Patients with the c.1179_1230del and c.1234_1235insTTGTC mutations have shown distinct clinical characteristics, but further research is required to confirm this result.

## Ethics

Ethics Committee Approval: The present study was approved by the Ethics Committee of Qilu Hospital, Shandong University (Jinan, China); Informed Consent: Written informed consent was obtained from all participants for treatment and the cryopreservation of bone marrow and peripheral blood according to the Declaration of Helsinki.

## Figures and Tables

**Table 1:**
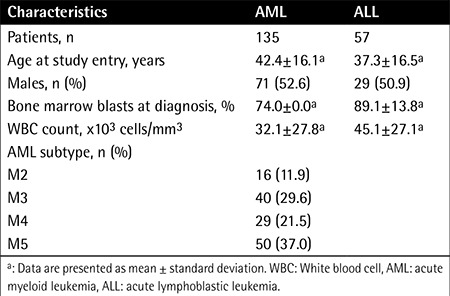
Clinical characteristics of 192 patients with acute leukemia.

**Table 2:**
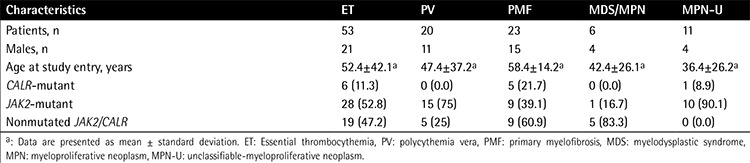
Clinical characteristics of 113 patients with myeloproliferative neoplasms.

**Table 3:**
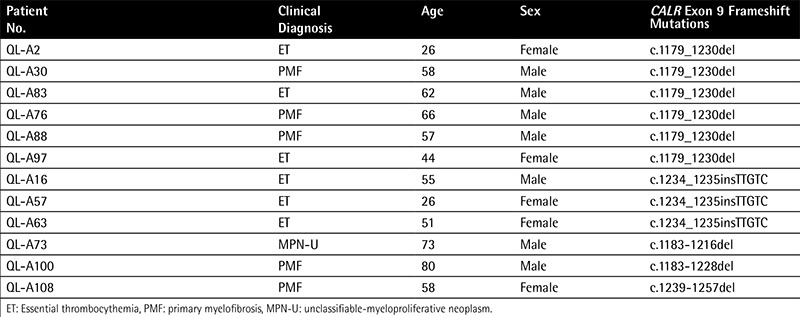
CALR exon 9 mutation profile in 12 myeloproliferative neoplasm patients.

**Table 4:**
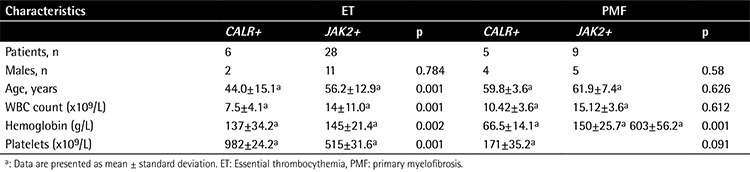
Clinical characteristics of essential thrombocythemia and primary myelofibrosis patients with CALR and JAK2 mutation.
